# Neural correlates of body comparison and weight estimation in weight-recovered anorexia nervosa: a functional magnetic resonance imaging study

**DOI:** 10.1186/s13030-018-0134-z

**Published:** 2018-10-31

**Authors:** Naoki Kodama, Yoshiya Moriguchi, Aya Takeda, Motonari Maeda, Tetsuya Ando, Hiroe Kikuchi, Motoharu Gondo, Hiroaki Adachi, Gen Komaki

**Affiliations:** 1 Division of Psychosomatic Medicine, Department of Neurology, University of Occupational and Environmental Health, Kitakyushu, 807-8555 Japan; 2 Department of Psychophysiology, National Institute of Mental Health, National Center of Neurology and Psychiatry, Tokyo, 187-8553 Japan; 3 NPO Corporation Nobinokai, Yokohama, 236-0014 Japan; 4 College of Art and Design, Joshibi University of Art and Design, Sagamihara, 252-8538 Japan; 5 Department of Psychosomatic Research, National Institute of Mental Health, National Center of Neurology and Psychiatry, Tokyo, 187-8553 Japan; 6 Department of Psychosomatic Medicine, Center Hospital of the National Center for Global Health and Medicine, Tokyo, 162-8655 Japan; 7 Department of Psychosomatic Medicine, Graduate School of Medical Sciences, Kyushu University, Fukuoka, 812-8582 Japan; 8 Department of Neurology, University of Occupational and Environmental Health, Kitakyushu, 807-8555 Japan; 9 School of Health Sciences Fukuoka, International University of Health and Welfare, Fukuoka, 831-8501 Japan

**Keywords:** Body dissatisfaction, Body image, Anorexia nervosa, Anterior cingulate cortex, Extrastriate body area, fMRI

## Abstract

**Background:**

The neural mechanisms underlying body dissatisfaction and emotional problems evoked by social comparisons in patients with anorexia nervosa (AN) are currently unclear. Here, we elucidate patterns of brain activation among recovered patients with AN (recAN) during body comparison and weight estimation with functional magnetic resonance imaging (fMRI).

**Methods:**

We used fMRI to examine 12 patients with recAN and 13 healthy controls while they performed body comparison and weight estimation tasks with images of underweight, healthy weight, and overweight female bodies. In the body comparison task, participants rated their anxiety levels while comparing their own body with the presented image. In the weight estimation task, participants estimated the weight of the body in the presented image. We used between-group region of interest (ROI) analyses of the blood oxygen level dependent (BOLD) signal to analyze differences in brain activation patterns between the groups. In addition, to investigate activation outside predetermined ROIs, we performed an exploratory whole-brain analysis to identify group differences.

**Results:**

We found that, compared to healthy controls, patients with recAN exhibited significantly greater activation in the pregenual anterior cingulate cortex (pgACC) when comparing their own bodies with images of underweight female bodies. In addition, we found that, compared with healthy controls, patients with recAN exhibited significantly smaller activation in the middle temporal gyrus corresponding to the extrastriate body area (EBA) when comparing their own bodies, irrespective of weight, during self-other comparisons of body shape.

**Conclusions:**

Our findings from a group of patients with recAN suggest that the pathology of AN may lie in an inability to regulate negative affect in response to body images via pgACC activation during body comparisons. The findings also suggest that altered body image processing in the brain persists even after recovery from AN.

**Electronic supplementary material:**

The online version of this article (10.1186/s13030-018-0134-z) contains supplementary material, which is available to authorized users.

## Background

Anorexia nervosa (AN) is a disorder of unknown etiology, mostly affecting young women. AN is characterized by immoderate food restriction, inappropriate eating habits, and distorted body image. The condition is associated with high rates of chronicity, morbidity, and mortality. However, the lack of understanding of the pathophysiology underlying AN has hindered the development of effective treatments.

Body dissatisfaction is a core pathophysiological feature of AN, manifesting as a negative subjective evaluation of the weight and shape of one’s own body. Indeed, body dissatisfaction is considered a diagnostic feature of AN, defined as “undue influence of weight and shape on self-evaluation” (American Psychiatric Association, 2013), and is considered an important factor in the development, maintenance, and relapse of AN [1–3].

The increasing prevalence of body dissatisfaction may be related to the sociocultural impact of the “thin ideal”, by which the concept of an idealized slim female body is promoted by mass media [4]. Current societal standards of beauty promote the thin ideal, which most women are unable to achieve [5]. Furthermore, research has consistently shown that the extent of exposure to mass media is positively correlated with body dissatisfaction [4]. Exposure to idealized female bodies via mass media is thought to prompt viewers to compare their own body with the ideal body shape, resulting in dissatisfaction with their own body and consequent emotional distress or anxiety, as described by Festinger’s Social Comparison Theory [6–8]. One study of patients with eating disorders (ED) revealed that the extent of body-related social comparison was strongly correlated with eating disorder symptoms [9], such that comparison of one’s own body with the thin ideal was a risk factor for the development of eating disorders.

One body shape comparison study reported that patients with AN exhibit greater activation of the right sensorimotor regions (insula and premotor cortex) and reduced activation of the pregenual ACC during self-other comparisons of body shape compared with healthy controls [10]. However, because idealized body images were the only visual stimuli tested, it is unclear whether participants compared themselves with the models in terms of slimness (i.e. social comparison) or if they were responding only to the “human body” component of the stimuli. This issue could be clarified by presenting a control condition not involving social comparisons by using additional body images, such as images of healthy weight and overweight bodies that would not be expected to trigger negative social comparison. Such an approach may clarify whether anxiety is triggered in patients by the social comparison of their own body image with others’ body image. Moreover, the effects of starvation or low body weight on cerebral blood flow in patients with AN presents another potentially confounding variable. This issue may be ameliorated by testing recovered patients with AN (recAN) [11, 12]. Patients with recAN are considered to have “normal-weight AN” in terms of the underlying cognitive pathophysiology and neural function. Patients with recAN are a normal-weight such that they have physiological healthy global cerebral blood flow [13], although they often continue to have modest but persistent dysphoric mood, obsessionality, body image concerns, and body dissatisfaction, as do patients with AN [14, 15]. However, the cognitive alterations in recAN are less severe than in patients with AN, such that the difference between recAN and AN is a matter of extent [14]. To the best of our knowledge, no previous studies have examined brain activity in patients with recAN during body-related social comparisons. Examining recovered patients may thus provide new insight into the process of recovery from AN.

In the present study, we used functional magnetic resonance imaging (fMRI) to investigate the neural correlates of body comparisons in recAN and control participants. We examined cerebral responses during the comparison between the self and images of others’ bodies, with visual stimuli depicting features of underweight, healthy weight, and overweight variants of a canonical female body, to reveal the specific impact of social comparison between the self and underweight female body images on brain responses in patients with recAN.

There are two components that cause body image distortion in AN: 1) perceptual disturbances and 2) dissatisfaction with one’s own body as a result of social comparison [16]. Accordingly, we used two experimental tasks to investigate each of the two disturbances. In the first, participants estimated the weight of a female body from a photograph (“weight estimation task”), which draws attention to others’ body shapes and weights. In the second, participants compared their own body shape with another female body in a photograph and were asked to rate their subjective anxiety level (“comparison task”). When perceiving images of bodies during the weight estimation task, patients with recAN are hypothesized to show reduced activity in brain systems concerned with processing body size/image than do healthy controls due to abnormalities in the visual processing of body size and shape. However, during the comparison task, we hypothesized that patients with recAN would report more anxiety and display higher activity in emotion-related brain regions. More specifically, we focused on areas that have been reported in previous research in which patients compared their own body with an idealized slim body shape [17]. These regions included the left pregenual ACC, right dorsolateral prefrontal, right inferior parietal lobule, right lateral fusiform gyrus, and left lateral fusiform gyrus [17]. In addition, we performed an exploratory whole-brain analysis to identify group differences.

## Methods

The study was approved by the local Ethics Committee (National Center of Neurology and Psychiatry) and conducted in accordance with the Declaration of Helsinki.

### Participants

We recruited 15 women who had recovered from AN (recAN), nine of whom recovered from the restricting subtype and six from the binge/purge subtype. We also recruited 14 healthy women who had never suffered from any ED (controls). One patient with recAN was excluded due to movement artifacts in the images, and one was excluded because of the presence of current AN symptoms. Another patient with recAN dropped out because of retraction of consent to participate. Finally, one control participant was excluded due to excessive image distortion within the frontal cortex caused by a hardware error. Consequently, the final analysis included 12 women with recAN (seven restricting and five binge/purge subtype) and 13 control participants. Two patients with recAN who recovered from the restricting subtype were receiving fluvoxamine. One with recAN who recovered from the restricting subtype was receiving sertraline. One with recAN who recovered from the binge/purge subtype was receiving paroxetine and etizolam. One with recAN who recovered from the binge/purge subtype was receiving fluvoxamine, ethyl loflazepate, and etizolam. One patient with recAN who recovered from the restricting subtype met the criteria for generalized anxiety disorder. One patient with recAN who recovered from the binge/purge subtype met the criteria for obsessive compulsive disorder. The two groups were matched in terms of age, education, and predicted IQ (Table 1).

**Table 1:** Comparisons of demographic and clinical characteristics between the patient and control groups

	Mean ± standard error		Student’s *t*	*p*-value
	Patients with recAN	Controls		
Age (years)	33.2 ± 0.08	29.7 ± 2.83	−1.00	0.33
BMI (kg/m^2^)	20.7 ± 0.71	21.5 ± 0.714	0.80	0.43
Education (years)	14.3 ± 0.71	14.3 ± 0.74	1.45	0.16
Predicted IQ	108.1 ± 2.73	109.1 ± 2.53	−0.27	0.78
Lowest past BMI (kg/m^2^)	12.9 ± 0.53	–	–	–
Age of onset (years)	17.6 ± 0.77	–	–	–
Length of recovery (years)	5.6 ± 1.19	–	–	–

*recAN* recovered anorexia nervosa, *BMI* body mass index

All women with recAN were recruited between February 2011 and August 2013 from *Nobino-Kai*, a non-governmental self-help support organization specializing in ED in Kanagawa, Japan. All recAN participants underwent two screening phases: (1) a brief phone screening with a clinical psychologist or medical doctor; and (2) a comprehensive assessment using a structured psychiatric interview (the Mini-International Neuropsychiatric Interview [MINI] [18, 19]) and a face-to-face interview and physical examination with a medical doctor with experience in ED. Control participants were recruited through our healthy participant database and public advertisements. Control participants had no history of an eating disorder or any psychiatric or serious medical or neurological illness, no first-degree relatives with an eating disorder, and had been within normal weight range since menarche.

Inclusion criteria for recAN included having previously met DSM-IV criteria for AN, with subsequent successful recovery from AN. The definition of recovery was no longer meeting the DSM-IV criteria for AN, including maintaining a weight above 85% of the standard body weight, having regular menstrual cycles, and not having engaged in binge eating, purging, or significant restrictive eating patterns for at least 1 year before the study. None of the participants met the exclusion criteria for alcohol or drug abuse or dependence, major depressive disorder, or severe anxiety disorder within 3 months before the experiment. General exclusion criteria included a history of head injury, hearing or visual impairment, any neurological disease, metallic implants or claustrophobia. Participants were asked to avoid eating and drinking caffeinated beverages for 2 h and alcohol for 24 h preceding the experiment.

Participants were informed that they were taking part in a study investigating the neural processing of body-related information. We asked participants who completed the experiment not to tell other participants anything about the experiment. Participants who completed the experiment left the experiment room without meeting other participants.

### Preparation of task stimuli

**Fig. 1:**
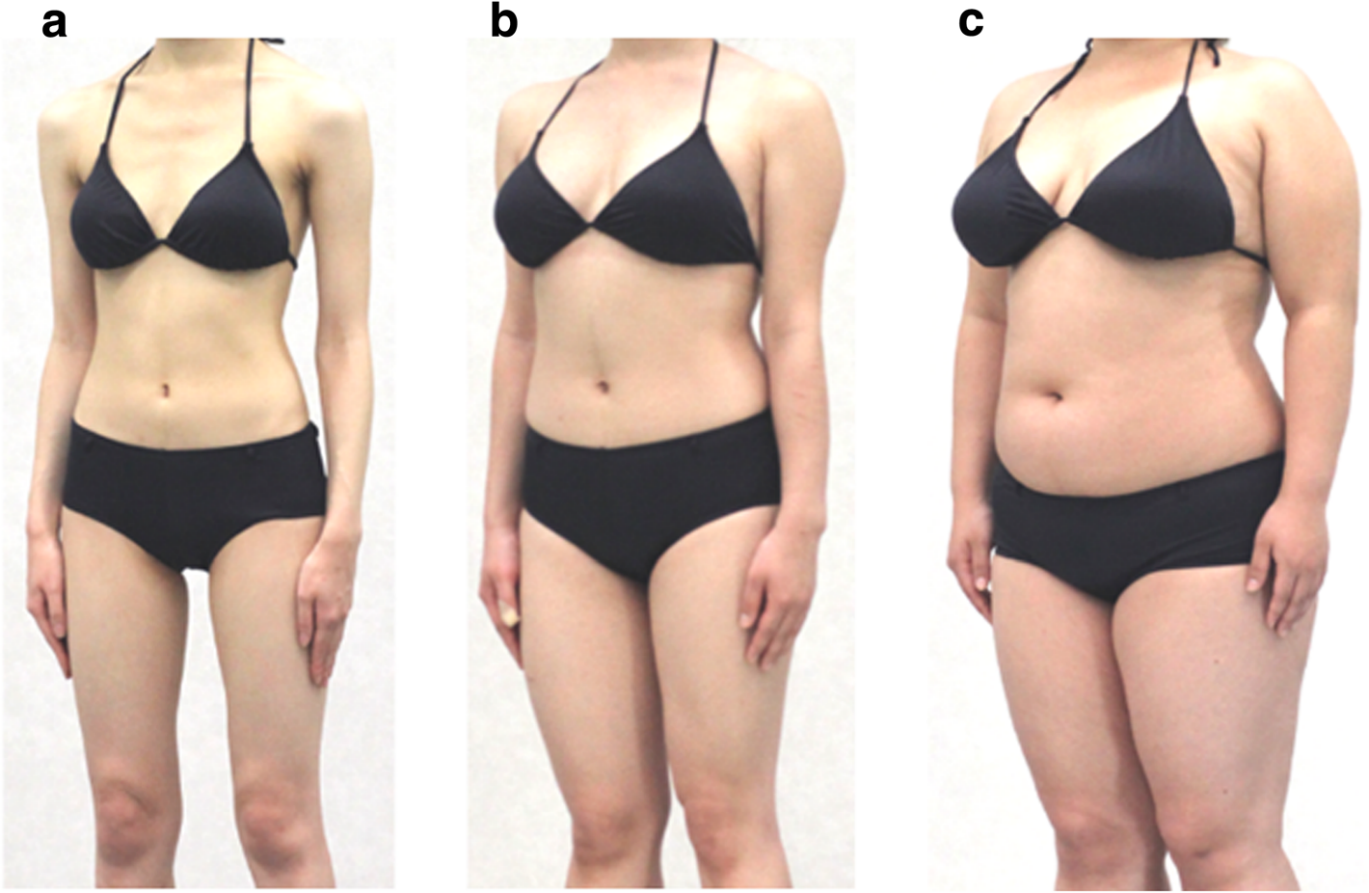
Examples of body images presented in the task during the fMRI scanning. We presented three types of photographs of female models with different body shapes: (**a**) underweight (41 kg, body mass index [BMI] 16 kg/m^2^); (**b**) healthy weight (54 kg, BMI 21 kg/m^2^); and (**c**) overweight (75 kg, BMI 29 kg/m^2^). The three models were the same age (21 years old) and height (160 cm)

We employed and took photographs of three female models who were 21 years old and 160 cm tall, but were within three different body weight categories (underweight: 41 kg, body mass index [BMI] 16 kg/m^2^; healthy weight: 54 kg, BMI 21 kg/m^2^; and overweight: 75 kg, BMI 29 kg/m^2^ [20, 21]. Example photographs are shown in Fig. 1. All photographs were taken in the same room under identical conditions, with the models wearing a uniform black bikini in front of a white wall. Each woman was photographed in two postures (placing the arms behind the head, or down beside the body) from 12 angles (in 30-degree increments) for each posture, from the neck down [22, 23]. Three sets of 24 photographs (72 photographs in total) of each model’s body were used as stimuli.

### fMRI parameters

MR images were acquired using a 1.5 T Siemens Magnetom Vision Plus System. To obtain functional imaging with a blood-oxygenation-level-dependent (BOLD) contrast, changes in the T2*-weighted MR signal were measured with a gradient echo-planar imaging (EPI) sequence (repetition time [TR] = 2500 ms, echo time [TE] = 40 ms, field of view [FOV] = 192 mm, flip angle = 90 degree, 64 × 64 matrix, 31 slices per slab, slice thickness = 3.5 mm, 1 mm gap along the AC-PC plane). We conducted four functional imaging runs to obtain 227 EPI volume images in each run, with the first five volumes discarded because of instability of magnetization. Thus, the final analysis included four sets of the remaining 222 EPI volumes in each run, resulting in 888 volumes.

### Experimental paradigm and procedure

**Fig. 2:**
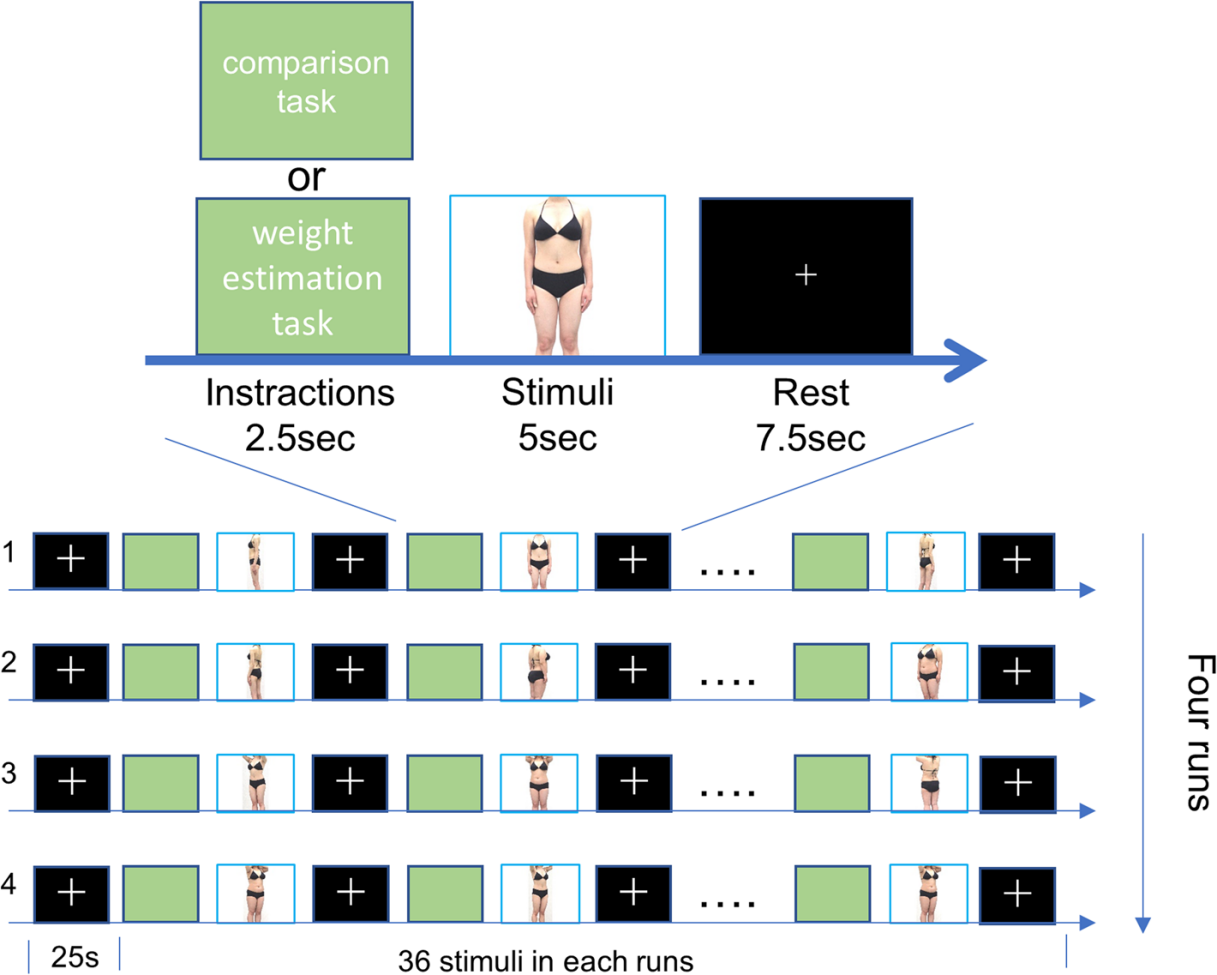
fMRI paradigm. We conducted four functional imaging runs. In each trial, participants were exposed to one body photograph presented on the screen, followed by either the “comparison task” or the “weight estimation task”. In the comparison task, participants were asked to compare their own body shape with the presented body photograph, and to rate their own subjective anxiety level in response to the photograph using four buttons on an MRI-compatible button pad (1 = “calm”, 2 = “somewhat calm”, 3 = “slightly anxious”, and 4 = “anxious”). In the weight estimation task, participants were required to objectively estimate the weight of the body in the photograph, selecting one of four weight categories (35, 55, 65, or 80 kg) using the 4-button pad. Each trial started by indicating the task with presentation of “comparison task” or “weight estimation task” for 2.5 s, followed by the task response, lasting for 5 s. Each trial ended with a fixation cross that continued to be presented during a 7.5 s inter-trial interval

In each functional imaging run, participants performed repeated event-related trials. In each trial, participants were exposed to one body photograph presented on a screen, followed by either the “comparison task” or the “weight estimation task”. In the comparison task, participants were asked to compare their own body shape with the presented body photograph, and to rate their own subjective anxiety level in response to the photograph using four buttons on an MRI-compatible button pad (1 = “calm”, 2 = “somewhat calm”, 3 = “slightly anxious”, and 4 = “anxious”). In the weight estimation task, participants were required to objectively estimate the weight of the body in the photograph, selecting one of four weight categories (35, 55, 65, or 80 kg) using the 4-button pad. Each trial started by indicating the task by displaying “comparison task” or “weight estimation task” for 2.5 s, followed by the task response, lasting for 5 s. Each trial ended with a fixation cross that continued to be presented during a 7.5 s inter-trial interval. The entire scanning procedure consisted of four separate runs, each containing 36 stimuli in a randomized order (Fig. 2). Instructions and body photographs were presented on a rear-projection screen, viewed via a mirror fitted to the MRI head coil. Response times from the start of the task to the button press were measured in each trial.

To measure anxiety levels more precisely in response to each photo in the comparison task, participants also rated the images after leaving the scanner. Participants were presented with each photo together with the following written question: “How do you feel when comparing your own body shape with this body image?” They responded on a 7-point scale for anxiety (1 = “very calm”, 2 = “calm”, 3 = “somewhat calm”, 4 = “neutral”, 5 = “slightly anxious”, 6 = “anxious”, or 7 = “very anxious”). To measure the accuracy of weight estimation in more detail, participants rated the photos used in the weight estimation task on a visual analogue scale (VAS), with a written question: “How much do you estimate the person’s body shown in the image weighs?” The VAS line ranged from 10 to 90 kg, with 10 kg increments.

Participants completed the following battery of self-report questionnaires: the Eating Disorder Examination Questionnaire (EDE-Q), an established assessment of behavioral and attitudinal eating pathology [24, 25]; the Eating Disorder Inventory-2 (EDI-2), a validated self-report instrument measuring eating problems [26, 27]; the 21-item Beck Depression Inventory Version 2 (BDI-2), a widely-used measure of psychological and physical symptoms of depression in adults [28, 29]; and the State-Trait Anxiety Inventory (STAI), a validated tool for evaluating anxiety including two dimensions (state anxiety (Y-1), which evaluates the emotional state of an individual in a particular situation, and trait anxiety (Y-2), which refers to a relatively stable personality characteristic [30, 31]). Finally, we employed the Japanese version of National Adult Reading Test (JART) [32] as a convenient alternative tool to measure participants’ IQ.

### fMRI data analyses

Image processing and statistical analyses were conducted with Statistical Parametric Mapping, version 12 (SPM12, Wellcome Department, London, UK https://www.fil.ion.ucl.ac.uk/spm/software/spm12/). Preprocessing of functional scans included realignment of functional images for motion correction using the first scan as a reference and spatial normalization to a standard template (Montreal Neurological Institute; MNI) with a resampled voxel size of 2 × 2 × 2 mm. Spatial smoothing with an 8 mm Gaussian kernel was applied to the analyzed images.

For first-level within-participant analysis, a design matrix for an individual general linear model (GLM) was constructed, incorporating six regressors corresponding to the 2 task types (comparison or weight estimation) × 3 types of weight (underweight, healthy weight, or overweight). The regressors were hypothetical hemodynamic responses modeled with the onset of corresponding events and convolved with a canonical hemodynamic response function. Intrinsic autocorrelations were accounted for by AR(1) and low frequency drift was removed via a high pass filter (128 s) in individual GLM analyses. The six 3D beta images including beta estimates from the GLM were fed into the subsequent second-level group analysis.

At the second level, a group analysis was performed using a full factorial design with one between-participants and one within-participants factor for testing the 2-way group (recAN or controls) × weight (underweight, healthy weight, or overweight) interaction effect during each of the comparison and weight-estimation tasks.

Our primary statistical analyses investigated weight-by-group interactions in constrained regions of interest (ROIs) during each of the comparison and weight-estimation tasks. We localized ROIs in the ACC, dorsolateral prefrontal, inferior parietal lobules, and lateral fusiform gyrus (FG), based on activations reported in a previous study of a body comparison task [17]. Each ROI was defined by a 12 mm radius sphere centered on the reported Montreal Neurological Institute (MNI) coordinates (left pregenual ACC: *x* = − 7, *y* = 34, *z* = 5; right dorsolateral prefrontal: *x* = 47, *y* = 2, *z* = 36; right inferior parietal lobule: *x* = 52, *y* = − 33, *z* = 42; right lateral fusiform gyrus: *x* = *x* = 47, *y* = − 57, *z* = − 15; left lateral fusiform gyrus: *x* = − 47, *y* = − 65, *z* = − 12). We created a single template for all ROIs using the MarsBaR version 0.44 (available at http://marsbar.sourceforge.net/) [33]. The small volume correction method was used to investigate the ROIs as a whole. We also performed voxel-by-voxel analyses within a whole set of ROIs, with the SVC method and the statistical significance level set at *p* < 0.05 corrected for family-wise error (FWE).

To investigate explanatory activation outside predetermined ROIs, we performed an exploratory whole-brain analysis to identify group differences, with a height threshold of *p* < 0.001 (uncorrected) and an extent threshold of *p* < 0.05 corrected for false discovery rate (FDR) [34].

### Statistical analysis

We compared continuous variables with Student’s *t*-tests. When necessary, *t*-tests were modified for unequal variances. For the variables from self-report questionnaires, we used Mann-Whitney *U* tests. Response times were analyzed with two-way repeated-measures ANOVAs (weight × group). Statistical analyses of non-imaging data were performed using IBM SPSS Statistics version 24 for Windows (IBM Corp., Chicago, USA), with statistical significance set at *p* < 0.05. We used Bonferroni corrections for the multiple comparisons analyses, such as the separate analyses for each stimulus weight.

## Results

### Baseline characteristics

There were no group differences in demographic variables (Table 1). Patients with recAN scored marginally higher on state anxiety (Y-1) than did controls, but the difference was not statistically significant. Patients with recAN scored significantly lower on body dissatisfaction in the EDI-2 and marginally lower on the drive for thinness in the EDI-2 compared with controls. Patients with recAN scored significantly higher on perfectionism than control participants in the EDI-2 (Table 2).

**Table 2:** Questionnaires characterizing participants’ inner body image and affect

	Mean ± standard error		Mann–Whitney U	*p*-value
	Patients with recAN	Controls		
EDE-Q score				
Restraint	0.2 ± 0.12	0.4 ± 0.18	56.0	0.38
Eating concern	0.6 ± 0.22	0.4 ± 0.14	65.0	0.71.
Weight concern	1.4 ± 0.34	1.4 ± 0.34	66.5	0.76
Shape concern	1.5 ± 0.34	1.7 ± 0.32	66.0	0.76.
EDI-2 score				
Drive for thinness	1.9 ± 0.74	4.7 ± 1.12	42.5	0.052†
Bulimia	2.1 ± 1.32	0.92 ± 0.47	74.0	0.85
Body dissatisfaction	5.5 ± 1.65	12.0 ± 1.94	35.5	0.02*
Ineffectiveness	9.0 ± 2.10	4.5 ± 0.89	48.5	0.11
Perfectionism	5.7 ± 0.95	1.9 ± 0.70	20.0	0.001**
Interpersonal distrust	4.9 ± 1.18	2.5 ± 0.77	48.0	0.11
Interoceptive awareness	3.7 ± 1.79	2.2 ± 0.52	76.0	0.94
Maturity fears	3.3 ± 0.87	4.6 ± 0.95	56.0	0.25.
BDI-II	11.0 ± 3.64	11.0 ± 3.40	73.5	0.81
STAI(Y-1)	45.0 ± 3.36	36.8 ± 2.23	44.5	0.07†
STAI(Y-2)	46.7 ± 3.13	42.6 ± 2.50	63.5	0.44

† *p* < 0.1 * *p* < 0.05 ** *p* < 0.01

*EDI-Q* Eating Disorder Examination Questionnaire, *EDI-2* Eating Disorder Inventory-2, *BDI-II* Beck Depression Inventory Version 2, *STAI(Y-1)* State-Trait Anxiety Inventory, state anxiety, *STAI(Y-2)* State-Trait Inventory, trait anxiety

These results indicated that patients with recAN no longer exhibited typical diagnostic symptomatology of AN, but still displayed significantly heightened perfectionism compared with the general population, even after long-term weight restoration and recovery [17, 35]. Interestingly, reported body dissatisfaction was lower in the recAN group, indicating that recovered patients were, at least subjectively, satisfied with their own body image when not involved in a social comparison situation.

### Behavioral results during the task

There were no significant group differences observed in overall response time (*F*(1, 23) = 0.447, *p* = 0.510, and *F*(1, 23) = 0.294, *p* = 0.593 for the comparison and weight estimation tasks, respectively). We also did not observe any significant group differences in anxiety levels on the 4-point scale during the comparison task in the scanner (*F*(1, 23) = 0.41, *p* = 0.84), and this result was confirmed on the 7-point scale for anxiety after scanning *(F*(1, 23) = 0.13, *p* = 0.725).

Compared with controls, patients with recAN marginally underestimated the weight of female body photos during scanning, but the difference did not reach statistical significance (*F*(1, 23) = 4.20, *p* = 0.052; Table 3). These results were also reflected by the VAS scale estimations after scanning (*F*(1, 23) = 0.001, *p* = 0.997). However, more detailed analysis revealed that patients with recAN underestimated the weights of underweight body photos (approximately 41 kg in reality) compared with controls. This was indicated by a significant weight × group interaction on body weight estimation (*F*(2, 46) = 8.02, *p* = 0.001; two-way repeated measures ANOVA), and by lower weight estimation values in recAN than in controls (recAN: mean ± SE = 39.2 ± 1.21 kg; controls: 42.5 ± 1.00 kg, *p* = 0.04, Bonferroni-corrected). Controls tended to underestimate the weight of overweight female body images (representing 75 kg) more than patients with recAN (recAN: mean ± SE = 67.7 ± 1.50 kg; controls: 64.3 ± 1.15 kg, *p* = 0.08; Table 4).

**Table 3:** Rating scores for the comparison task and weight estimation task

	Mean ± standard error		*F* value (1, 23)	*p*-value
	Patients with recAN	Controls		
Comparison task			0.41438	0.84
Underweight	2.1 ± 0.26	1.6 ± 0.22		0.22
Healthy weight	2.1 ± 0.19	2.0 ± 0.20		0.82
Overweight	3.3 ± 0.07	3.4 ± 0.09		0.11
Weight estimation task			4.2022	0.052†
Underweight	1.1 ± 0.08	1.3 ± 0.10		0.12
Healthy weight	2.0 ± 0.02	2.2 ± 0.04		0.03*
Overweight	3.3 ± 0.07	3.4 ± 0.09		0.25

† *p* < 0.1 * *p* < 0.05 ** *p* < 0.01

Two-way repeated-measures ANOVA was used for group comparisons

Post-hoc *p*-values for ANOVA analyses were Bonferroni corrected

**Table 4:** 7-point scale for anxiety and VAS scores for weight estimation

	Mean ± standard error		*F* value (1, 23)	*p*-value
	Patients with recAN	Controls		
Comparison task			0.127097	0.725
Underweight	4.2 ± 0.52	4.6 ± 0.41		0.61
Healthy weight	3.7 ± 0.37	3.7 ± 0.38		0.97
Overweight	3.4 ± 0.32	2.6 ± 0.36		0.124
Weight estimation task (kg)^a^			0.000870	0.98
Underweight	39.2 ± 1.21	42.5 ± 1.0		0.04*
Healthy weight	52.0 ± 0.53	52.2 ± 0.85		0.88
Overweight	67.7 ± 1.49	64.3 ± 1.15		0.08†

† *p* < 0.1 * *p* < 0.05 ** *p* < 0.01

The two-way repeated-measures ANOVA was used for group comparisons

Post-hoc *p*-values for ANOVA analyses were Bonferroni corrected

^a^There was a significant interaction effect of weight × group (*F*(2, 46) = 8.020, *p* = 0.001028) for the weight estimation task

In summary, the two groups showed similar subjectively reported anxiety levels during self-other body shape comparison. However, estimation of other’s body weight differed between recAN patients and controls. There were no differences in behavioral results between the patients with past restricting AN and those with past binge/purging AN.

### fMRI results

#### ROI analyses

**Fig. 3:**
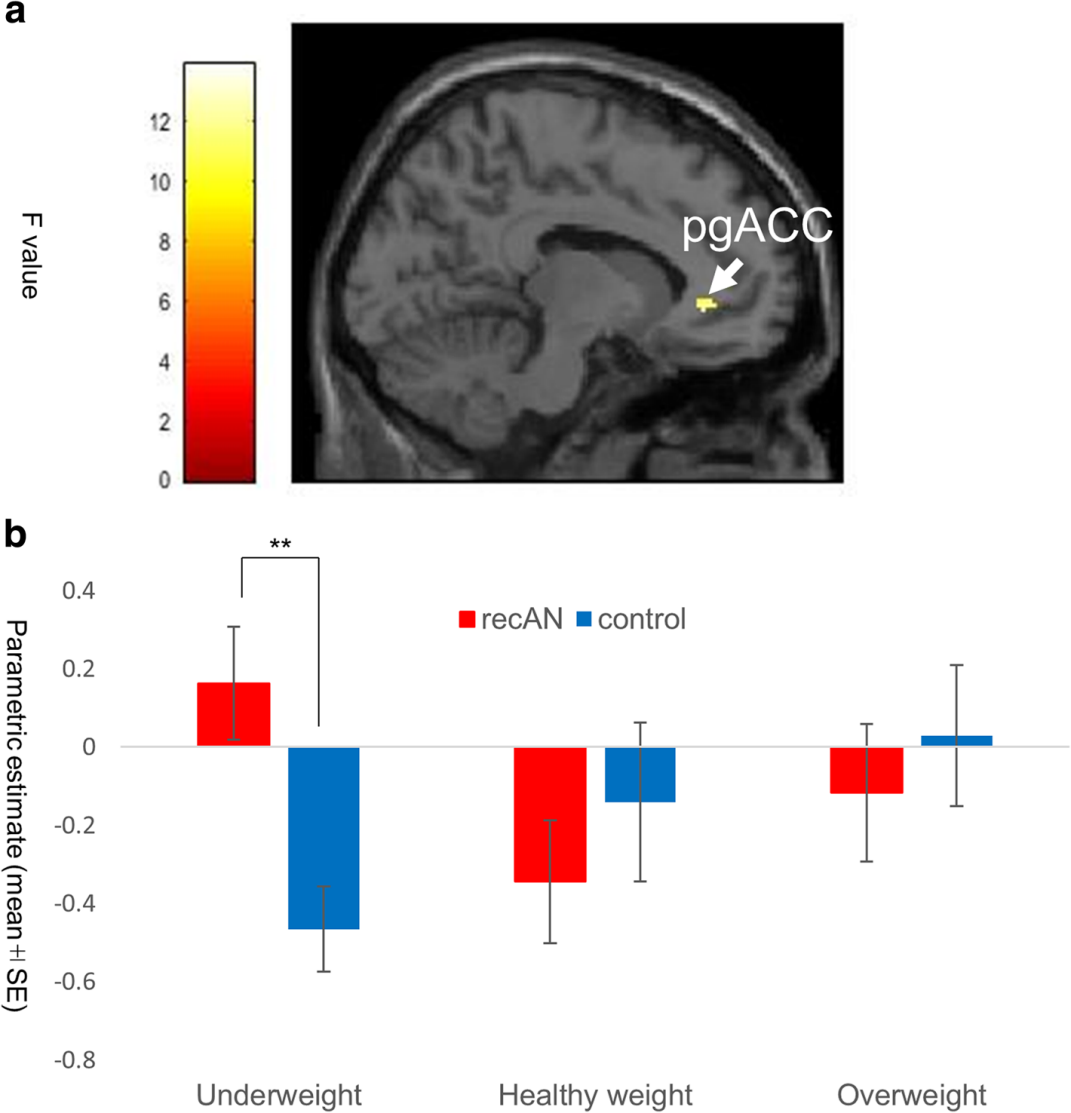
Group-by-stimulus 2-way interactive effect on BOLD signals during the comparison task. **a** The cluster rendered on the sagittal brain section shows the group-by-stimulus 2-way ANOVA interactive effect for the comparison task on BOLD signals in the left pregenual anterior cingulate (pgACC; the maximum peak of the effect at the MNI coordinates: *x* = − 10, *y* = 36, *z* = − 2 mm; cluster size: *k* = 6 voxels; peak *Z* score = 4.30, *p* = 0.008, FWE corrected for multiple comparisons). The analysis was based on a region of interest (ROI) analysis, with small volume correction (SVC) within the left pregenual ACC (a 12-mm radius sphere centered on *x* = − 7, *y* = 34, *z* = 5). The left color bar indicates the *F* value of the rendered cluster. **b** The bar graph shows the parametric estimates from the ANOVA analysis of the BOLD signals in the left pgACC cluster in each group (recAN and control) for each stimulus type (underweight, healthy weight, and overweight). Post hoc *t*-tests showed significantly greater BOLD signals in patients with recAN than in controls. Error bars indicate standard error. * *p* < 0.05 (Bonferroni-corrected), ** *p* < 0.01 (Bonferroni-corrected).. MNI: Montreal Neurological Institute; BOLD: blood oxygen level dependent; FWE: family-wise error; recAN: recovered anorexia nervosa

We observed a significant weight × group interaction effect during the comparison task in the left pregenual ACC (peak cluster coordinate: *x* = − 10, *y* = 36, *z* = − 2; cluster size: *k* = 6 voxels; *F*(2, 138) = 13.87, *p* = 0.008, FWE; Fig. 3, Additional file 1: Tables S1-S3) such that patients with recAN showed greater pregenual ACC activation in response to underweight images compared with controls (*T* = 3.47, *p* = 0.002, thresholded by Bonferroni correction).

In contrast, we did not observe a weight × group interaction effect during the weight estimation task on activation in any ROI.

#### Whole brain analysis

**Fig. 4:**
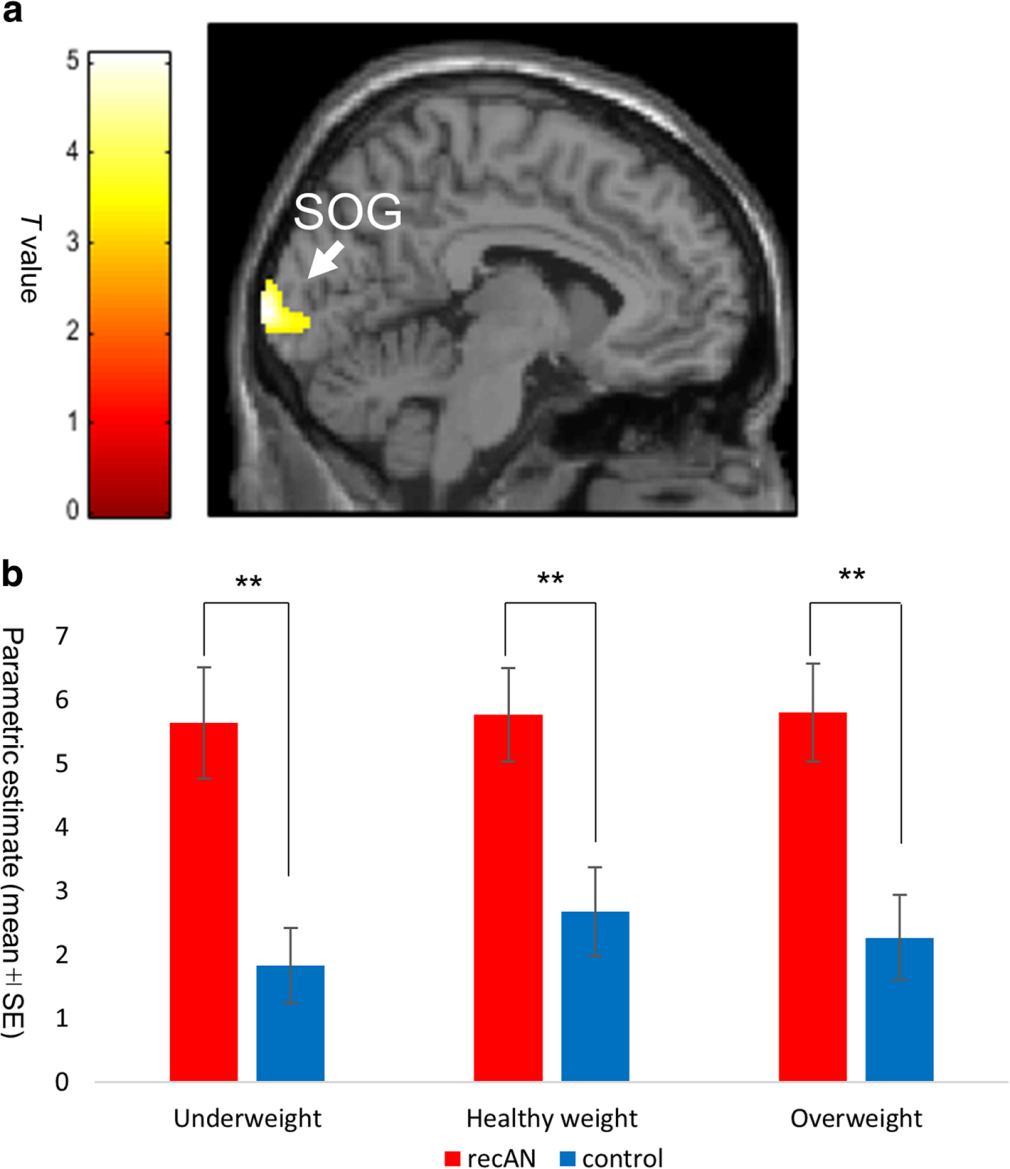
Group comparison (recAN > controls) of the brain BOLD signals during the comparison task. **a** The cluster rendered on the sagittal brain section shows the group effect (recAN > controls) in the comparison task on BOLD signals in the left visual cortex (left superior occipital gyrus). Maximum peak of the effect at the MNI coordinates: *x* = − 6, *y* = − 106, *z* = 4 mm; cluster size: *k* = 362 voxels; peak *T* score = 5.07, height level: *p* = 1.6 × 10^− 6^ (uncorrected), cluster level: *p* = 0.033; FDR corrected for multiple comparisons. The analyses were on a whole brain exploratory voxel-by-voxel basis. The left color bar indicates the *T* value of the rendered cluster. **b** The bar graph shows the parametric estimates of the BOLD signals in the left SOG cluster in each group (recAN and controls) for each stimulus type (underweight, healthy weight, and overweight). Post hoc *t*-tests showed significantly greater BOLD signals in patients with recAN than in controls, regardless of the stimulus types. Error bars indicate standard error. * *p* < 0.05, ** *p* < 0.01 (Bonferroni-corrected). MNI: Montreal Neurological Institute; BOLD: blood oxygen level dependent; FDR: false discovery rate; SOG, superior occipital gyrus; recAN: recovered anorexia nervosa

In addition, we explored group differences in whole brain responses to all body images (irrespective of weight) during the comparison and weight-estimation tasks. The patients with recAN exhibited greater visual cortex activation than did control participants during the comparison task (left superior occipital gyrus [BA17]; peak coordinate *x* = − 6, *y* = − 106, *z* = 4, *T* = 5.07, peak level *p* = 0.0000016 (uncorrected), cluster level *p* = 0.033 (FDR); Fig. 4, Additional file 1: Tables S1-S3). These results suggest that patients with recAN exhibit heightened attention to other’s body shapes, irrespective of weight during self-other comparisons of body shape. We also conducted post-hoc Bonferroni corrected T tests to examine the difference between those with a history of restricting and binge/purging AN. Patients with recAN who recovered from restricting AN (recANr) exhibited greater visual cortex activation than did patients with recAN who recovered from binge/purging AN (recANbp) (parametric estimates recANr: mean ± SE = 6.77 ± 0.90, recANbp: mean ± SE = 4.29 ± 1.07 *T* = 2.94, *p* = 0.008). When we analyzed separately for each weight group (underweight, healthy weight, or overweight), there was no significant difference between those with a history of restricting AN and those with a history of binge/purging AN.

**Fig. 5:**
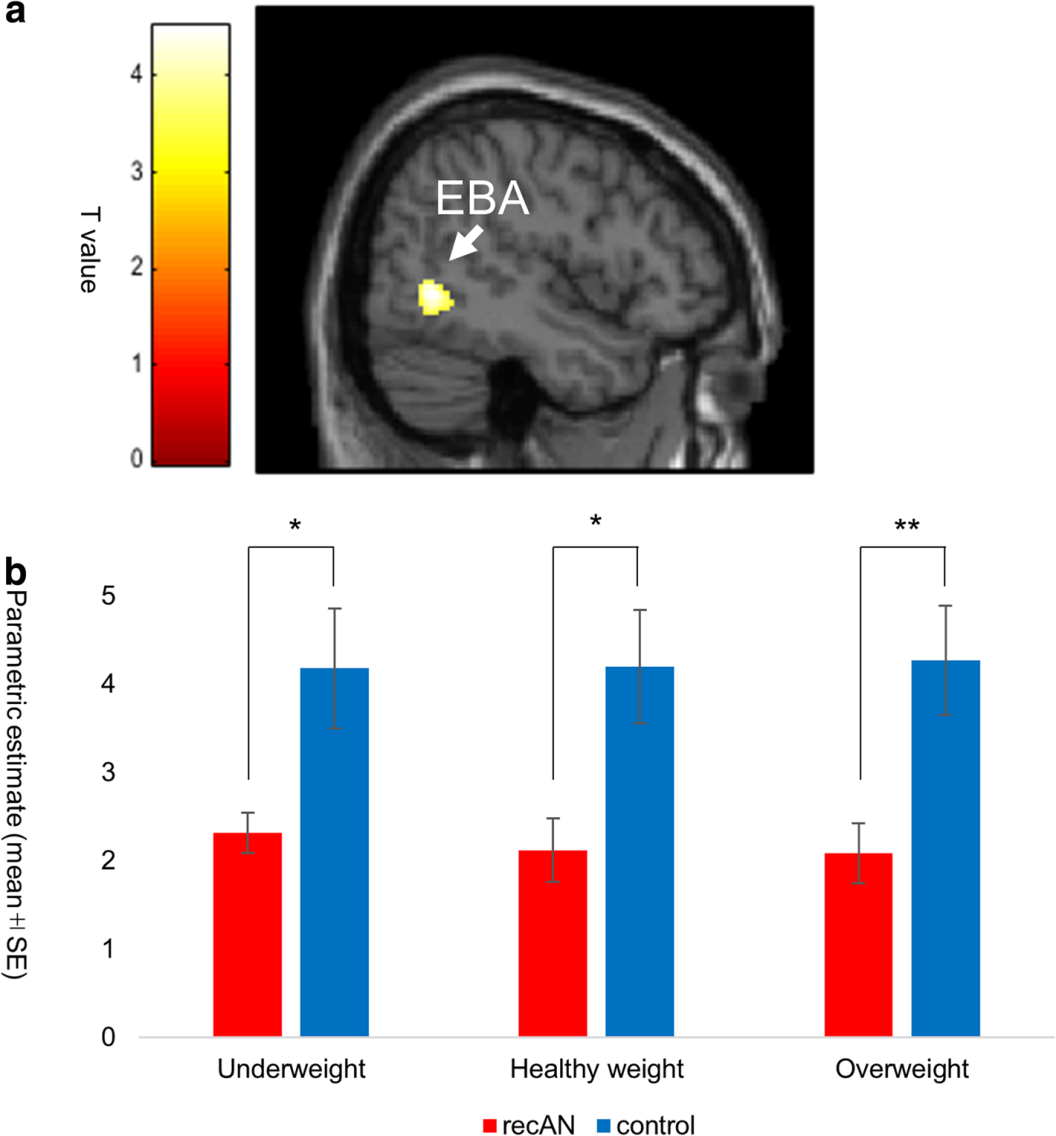
Group comparison (controls > recAN) of the brain BOLD signals during the weight estimation task. **a** The cluster rendered on the sagittal brain section shows the group effect (control > recAN) in the weight estimation task on BOLD signals in the middle temporal gyrus corresponding to the extrastriate body area (EBA, Brodmann’s area 37). The maximum peak of the effect at the MNI coordinates: *x* = 48, *y* = − 58, *z* = − 2 mm; cluster size: *k* = 131 voxels; peak *T* score *=* 4.49, height level: *p* = 1.39 × 10^− 5^ (uncorrected), cluster level: *p* = 0.029; FDR corrected for multiple comparisons. The analyses were on a whole brain exploratory voxel-by-voxel basis. The left color bar indicates the *T* value of the rendered cluster. **b** The bar graph shows the parametric estimates of the BOLD signals in the right EBA cluster in each group (recAN and controls) for each stimulus type (underweight, healthy weight, and overweight). Post hoc *t*-tests showed significantly greater BOLD signals in controls than in patients with recAN, regardless of the stimulus types. Error bars indicate standard error. * *p* < 0.05, ** *p* < 0.01 (Bonferroni-corrected). MNI: Montreal Neurological Institute; BOLD: blood oxygen level dependent; FDR: false discovery rate; recAN: recovered anorexia nervosa

In contrast, patients with recAN exhibited weaker activation during weight estimation in the middle temporal gyrus ([BA37]; peak coordinate *x* = 48, *y* = − 58, *z* = − 2, *T =* 4.49*,* peak level *p =* 0.0000139 (uncorrected), cluster level *p* = 0.029 (FDR), corresponding to the extrastriate body area, EBA [36]; Fig. 5, Additonal file 1: Table S4). This suggests that patients with recAN exhibited altered visual processing specific to human bodies.

## Discussion

In the current study, we investigated the neural underpinnings of body shape comparisons and weight estimation in patients with recAN. Our fMRI results identified differential neural correlates of self-other comparisons of body shape between patients with recAN and controls.

Our hypothesis was supported by the result that patients with recAN showed reduced activation of the right EBA during the weight estimation task. A potential advantage of our study is that patients with recAN of a healthy weight were not affected by structural cortical volume loss, which is evident in women suffering from ongoing AN, in the left lateral occipital cortex including the EBA [37]. Nevertheless, the current results seem consistent with a previous report that women with ongoing AN exhibited reduced EBA activation [38] and reduced effective connectivity with the fusiform body area [39]. The EBA does not respond to objects or parts of objects, but to human bodies and body parts such as hands and feet [36, 40, 41]. The decrease in EBA activity in patients with recAN suggests that altered neural processing of body information persists in patients who have had AN even after long-term recovery from the disorder (approximately 5–6 years). This may underlie the altered perception of others’ body shapes, and may be related to the high relapse rate (approximately 35%) in patients with recAN [42, 43].

Contrary to our hypothesis, there was no difference in anxiety levels during body shape comparisons. However, we observed that patients with recAN exhibited greater pregenual ACC activation than did controls during comparisons of their own body with images of an underweight body. The pregenual ACC is involved in detecting conflict in the emotional domain and recruiting cognitive control processes to resolve such conflict [44] [45]. A study of a similar self-other body-shape comparison task reported that healthy women exhibited activation in the left ACC [17], while patients with current AN exhibited reduced activation in the pregenual ACC [10]. A possible reason for the difference between our finding and those from previous reports is that recovery from AN may involve the recovery of top-down control of negative emotional impact during self-other comparison of body images, which may cause compensatory hyperfunction in the pregenual ACC.

At baseline, we observed that body dissatisfaction was lower among patients with recAN than among control participants, while anxiety levels during the self-other body comparison task did not differ between the groups. The significantly lower level of body dissatisfaction in recAN compared with controls appears to differ from findings reported in previous studies [35] [46]. However, this finding might be in accord with previous reports that weight regain is accompanied by significant reductions in body dissatisfaction. In our study, body dissatisfaction in recAN was even lower than that of healthy controls, although body dissatisfaction of recAN has been reported to be comparable to that of healthy controls [47]. This might partially be due to cultural background in a Japanese sample, such as the presence of higher body dissatisfaction in Japanese healthy women than in women from other countries (e.g., [48]). Such cultural differences may result in inconsistencies between findings from Japan and Western countries. Nonetheless, this is a matter of speculation and remains to be clarified. We observed that patients with recAN exhibited heightened perfectionism, consistent with previous reports that perfectionism persists among patients with AN who have achieved long-term weight restoration [35, 49–51] and was a risk factor for recurrence [52, 53]. Our results confirm the persistence of heightened perfectionism in patients with recAN.

During the comparison task, patients with recAN showed greater visual cortex activation, which was likely due to heightened attention in these women compared with controls. It is well established that sustained spatial attention modulates cortical responses in V1 [54, 55]. Heightened brain responses may be associated with exaggerated visual processing of body shapes in AN, given that one of the core symptoms of ED is obsessive preoccupation with body weight and shape. Moreover, patients with recANr showed greater activation of the visual cortex than did those with recANbp. This is consistent with a previous study reporting that patients with restricting AN had a larger attentional bias to threat word stimuli related to ED (e.g., FAT) than did healthy controls, whereas patients with binge-purging AN showed avoidance of threat word stimuli related to ED [56]. Our result suggests that differential patterns of attention allocation between patients with restricting AN and those with binge purging AN may remain even after recovery.

Several limitations should be considered when interpreting our results. The small number of participants is an obvious limitation, which should be addressed in future studies with larger sample sizes. In addition, the current study only measured subjective ratings, without simultaneous objective measures of arousal or anxiety, such as galvanic skin responses or papillary responses representing autonomic nervous activity. Thus, participants may have become habituated with repeated exposure to similar body image stimuli, potentially reducing signal intensity, as reported in previous imaging studies using fear and threat cues [57, 58]. Our discussion of AN is based on the assumption that there are certain similarities between recAN and AN. This may be a limitation of the study because recAN is not completely the same as AN. However, despite the differences between recAN and AN (mostly in physiological and nutrition status) the two have many similarities, especially in terms of psychological disturbances, which are likely persistent across the lifespan. For example, patients with AN show personality traits characteristic of AN (such as obsessive-compulsive personality) even before the onset of illness [52, 53], suggesting that such traits may derive from underlying genetic vulnerabilities. Furthermore, previous studies have reported that core temperament and personality traits persist after recovery from AN [35, 59], possibly due to the genetic background of those with the condition. Although we should be aware that recAN is not completely the same as AN, we believe that we can still discuss disturbances of psychological traits in AN based on the similarities between the two. Nonetheless, similar studies with patients with ongoing AN are warranted in the future. Finally, including a patient sample with current AN would enable direct comparison between patients with AN and recAN. Future studies comparing patients with AN and recAN may provide further insight into the recovery process.

## Conclusions

The current study revealed that patients with recAN exhibited greater pregenual ACC activation than did controls during comparisons of their own body with underweight female body images. These findings suggest that recovery from AN may involve the regulation of negative affect in response to body images via the pregenual ACC when a patient compares their own body with idealized underweight body images. In addition, there was reduced right EBA activity in patients with recAN, indicating that altered body image processing in the brain can persist even after recovery from AN.

## Additional file

Additional file 1: **Table S1.** Correlations (Spearman’s rho) between subjective anxiety ratings (7-point scale) and neural activation in response to the comparison task. **Table S2.** Correlations (Spearman’s rho) between body dissatisfaction assessed with EDI-2 scores and neural activation in response to the comparison task. **Table S3.** Correlations (Spearman’s rho) between perfection assessed with EDI-2 scores and neural activation in response to the comparison task. **Table S4.** Correlations (Spearman’s rho) between body dissatisfaction assessed with EDI-2 scores and neural activation in response to the weight estimation task. **Table S5.** Correlations (Spearman’s rho) between perfection assessed with EDI-2 scores and neural activation in response to the weight estimation task. (DOCX 25 kb)
