# Supplementary material for: Neural correlates of body comparison and weight estimation in weight-recovered anorexia nervosa: a functional magnetic resonance imaging study
Source: Biopsychosoc Med. 2018 Oct 31;12:15. doi: 10.1186/s13030-018-0134-z (PMC6208027; doi:10.1186/s13030-018-0134-z)
Supplement: Supplementary file 1 — Table S1. Correlations (Spearman’s rho) between subjective anxiety ratings (7-point scale) and neural activation in response to the comparison task. Table S2. Correlations (Spearman’s rho) between body dissatisfaction assessed with EDI-2 scores and neural activation in response to the comparison task. Table S3. Correlations (Spearman’s rho) between perfection assessed with EDI-2 scores and neural activation in response to the comparison task. Table S4. Correlations (Spearman’s rho) between body dissatisfaction assessed with EDI-2 scores and neural activation in response to the weight estimation task. Table S5. Correlations (Spearman’s rho) between perfection assessed with EDI-2 scores and neural activation in response to the weight estimation task. (DOCX 25 kb) [file 13030_2018_134_MOESM1_ESM.docx]

**Correlational analysis**

To investigate associations between neural activation and psychometric measures (the ratings of the anxiety level~~s~~, body-dissatisfaction, ~~the~~ and perfection ~~of~~ from the EDI-2 ~~scores~~), we extracted average parameter estimates (betas) from clusters obtained from ROI analyses and whole-brain analysis for each participant in the respective weight categories (underweight, healthy weight, or overweight) and participant groups (recAN or controls). Associations between average parameter estimates and psychometric measures were evaluated with Spearman’s (rho) correlation coefﬁcient and Bonferroni’s correction was applied for multiple comparisons, with statistical significance set at *p* < 0.05.

There was no significant correlation between neural activation and any psychometric ~~any~~ measure (see tables below). This may be due to insufficient sensitivity resulting from the small sample size, or due to experimental problems that are difficult to interpret.

**Table S1.** Correlations (Spearman’s rho) between subjective anxiety ratings (7-point scale) and neural activation in response to the comparison task

| Region | Group | Stimulus | Rho | Bonferroni corrected *p* |
| --- | --- | --- | --- | --- |
| Pregenual ACC | Control | Underweight | 0.069 | 1 |
|  |  | Healthy weight | -0.082 | 1 |
|  |  | Overweight | -0.256 | 1 |
|  | RecAN | Underweight | 0.350 | 0.792 |
|  |  | Healthy weight | -0.508 | 0.276 |
|  |  | Overweight | -0.522 | 0.246 |
| SOG | Control | Underweight | 0.311 | 0.903 |
|  |  | Healthy weight | 0.181 | 1 |
|  |  | Overweight | -0.228 | 1 |
|  | RecAN | Underweight | -0.462 | 0.39 |
|  |  | Healthy weight | -0.165 | 1 |
|  |  | Overweight | -0.620 | 0.096 |

ACC, anterior cingulate cortex; SOG, superior occipital gyrus

**Table S2.** Correlations (Spearman’s rho) between body dissatisfaction assessed with EDI-2 scores and neural activation in response to the comparison task

| Region | Group | Stimuli | Rho | Bonferroni corrected *p* |
| --- | --- | --- | --- | --- |
| Pregenual ACC | Control | Underweight | -0.074 | 1 |
|  |  | Healthy weight | -0.061 | 1 |
|  |  | Overweight | 0.078 | 1 |
|  | RecAN | Underweight | 0.487 | 0.326 |
|  |  | Healthy weight | 0.046 | 1 |
|  |  | Overweight | 0.116 | 1 |
| SOG | Control | Underweight | 0.050 | 1 |
|  |  | Healthy weight | -0.044 | 1 |
|  |  | Overweight | 0.053 | 1 |
|  | RecAN | Underweight | -0.011 | 1 |
|  |  | Healthy weight | -0.032 | 1 |
|  |  | Overweight | -0.011 | 1 |

EDI-2, Eating Disorder Inventory-2; ACC, anterior cingulate cortex; SOG, superior occipital gyrus

**Table S3.** Correlations (Spearman’s rho) between perfection assessed with EDI-2 scores and neural activation in response to the comparison task

| Region | Group | Stimuli | Rho | Bonferroni corrected *p* |
| --- | --- | --- | --- | --- |
| Pregenual ACC | Control | Underweight | -0.009 | 1 |
|  |  | Healthy weight | 0.005 | 1 |
|  |  | Overweight | 0.120 | 1 |
|  | RecAN | Underweight | 0.099 | 1 |
|  |  | Healthy weight | 0.325 | 1 |
|  |  | Overweight | -0.272 | 1 |
| SOG | Control | Underweight | -0.009 | 1 |
|  |  | Healthy weight | 0.034 | 1 |
|  |  | Overweight | -0.071 | 1 |
|  | RecAN | Underweight | -0.403 | 0.582 |
|  |  | Healthy weight | -0.389 | 0.636 |
|  |  | Overweight | -0.431 | 0.486 |

EDI-2, Eating Disorder Inventory-2; ACC, anterior cingulate cortex; SOG, superior occipital gyrus

**Table S4.** Correlations (Spearman’s rho) between body dissatisfaction assessed with EDI-2 scores and neural activation in response to the weight estimation task

| Region | Group | Stimuli | Rho | Bonferroni corrected *p* |
| --- | --- | --- | --- | --- |
| EBA | Control | Underweight | -0.232 | 1 |
|  |  | Healthy weight | -0.257 | 1 |
|  |  | Overweight | -0.127 | 1 |
|  | RecAN | Underweight | 0.042 | 1 |
|  |  | Healthy weight | -0.247 | 1 |
|  |  | Overweight | -0.007 | 1 |

EBA: extrastriate body area

**Table S5.** Correlations (Spearman’s rho) between perfection assessed with EDI-2 scores and neural activation in response to the weight estimation task

| Region | Group | Stimuli | Rho | Bonferroni corrected *p* |
| --- | --- | --- | --- | --- |
| EBA | Control | Underweight | 0.236 | 1 |
|  |  | Healthy weight | 0.273 | 1 |
|  |  | Overweight | 0.467 | 0.324 |
|  | RecAN | Underweight | 0.046 | 1 |
|  |  | Healthy weight | -0.201 | 1 |
|  |  | Overweight | -0.230 | 1 |

EBA: extrastriate body area
